# Surgical techniques and functional evaluation for vestibular lesions in the mouse: unilateral labyrinthectomy (UL) and unilateral vestibular neurectomy (UVN)

**DOI:** 10.1007/s00415-020-09960-8

**Published:** 2020-06-17

**Authors:** François Simon, David Pericat, Cassandre Djian, Desdemona Fricker, Françoise Denoyelle, Mathieu Beraneck

**Affiliations:** 1 CNRS, Integrative Neuroscience and Cognition Center, Université de Paris, 75006 Paris, France; 2 Department of Paediatric Otolaryngology, Hôpital Necker-Enfants Malades, AP-HP.Centre, Université de Paris, 75015 Paris, France; 3 Laboratoire de Neurosciences Sensorielles et Cognitives, CNRS, UMR 7260, Aix Marseille Université, 13331 Marseille, France

**Keywords:** Unilateral vestibular neurectomy, Unilateral labyrinthectomy, Mouse, Vestibular compensation, Surgery

## Abstract

**Objective:**

Unilateral labyrinthectomy (UL) and unilateral vestibular neurectomy (UVN) are two surgical methods to produce vestibular lesions in the mouse. The objective of this study was to describe the surgical technique of both methods, and compare functional compensation using vestibulo-ocular reflex-based tests.

**Methods:**

UL and UVN were each performed on groups of seven and ten mice, respectively. Main surgical landmarks were the facial nerve, the external auditory canal and the sternomastoid and digastric muscles. For UL, the sternomastoid muscle was elevated to expose the mastoid, which was drilled to destroy the labyrinth. For UVN, the bulla was drilled opened and a transcochlear approach enabled the identification of the vestibulo-cochlear nerve exiting the brainstem, which was sectioned and the ganglion of Scarpa suctioned. Behaviour and vestibular function were analysed before surgery and at 1, 4, 7 days and at 1 month postlesion using sinusoidal rotation, off-vertical axis rotation, static head tilts and angular velocity steps.

**Results:**

UL is a faster and safer procedure than UVN (operative time 16.3 vs 20.5 min, *p* = 0.19; survival rate 86% vs 60%, *p* = 0.25). UVN was more severe with significantly worse behavioural scores at day 4 and day 7 (*p* < 0.001). Vestibular compensation was overall similar during the first week and at 1 month (non-statistically significant difference).

**Conclusion:**

Both UL and UVN procedures can routinely be performed in the mouse with similar post-operative recovery and behavioural compensation. The operative risk of vascular or neurological damage is smaller in UL compared to UVN. UVN may be required for specific research protocols studying central cellular process specifically related to the destruction of the ganglion of Scarpa and following vestibular nerve degeneration.

**Electronic supplementary material:**

The online version of this article (10.1007/s00415-020-09960-8) contains supplementary material, which is available to authorized users.

## Introduction

The first description by Flourens in 1824 of the behavioural symptoms that follow an inner ear lesion is a starting point in the field of vestibular research [1]. The study of vestibular lesions and compensation in different vertebrate species has helped to understand vestibular physiology and the capacity of the brain to cope with the loss of a sensory function [2–10]. Unilateral labyrinthectomy (UL) and unilateral vestibular neurectomy (UVN) are two surgical methods to produce vestibular lesions. These are more invasive than pharmacological methods but are also more radical and definite lesions which may be required in certain research protocols [11]. Surgical techniques need to be described in detail to be reproducible [12–18]. Some articles report the surgical methods for UL or UVN in other animal models (e.g. frogs [19], chicks [20], rats [21–23], or cats [24–26]) but not in the mouse. Yet mouse models are currently the most used worldwide, due to ease of use for genetic engineering or breeding, and ethical limitations when working with larger mammals. Surgery in the mouse is challenging due to its small size and specific anatomy. Here we provide a detailed step-by-step description of both UL and UVN in adult mice. Video-oculography was used to assess vestibular function following successful surgery, and to monitor vestibular compensation by quantitative measurements of the vestibulo-ocular reflex [11, 14, 27]. The objective of this study was an update of existing protocols for vestibular lesions, allowing the investigator to improve their effectiveness and safety, and reduce the duration of the surgery, critical for survival. We discuss differences in the two approaches to help choose between UL and UVN as adequate methods for a vestibular lesion.

## Materials and methods

### Ethics statement

A total of 17 male C57/BL6J mice, aged 10–30 weeks, was operated on, seven mice for UL and ten for UVN. Animals were used in accordance with the European Communities Council Directive 2010/63/EU. All efforts were made to minimize suffering and reduce the number of animals included in the study. All procedures were approved by the ethical committee for animal research of the University of Paris (CEEA.34).

### Anaesthesia and peri-operative care

Anaesthesia and peri-operative care were identical for either UL or UVN procedure. A stock solution of anaesthesia was prepared including 1 ml Ketamine 100 mg/ml (Virbac, Carros, France), 500 μl Rompun^®^ 2% (Bayer Vital GmbH, Leverkusen, Germany), and 8.5 ml physiological saline solution. A dosage of 100 mg/kg ketamine and 10 mg/kg xylazine, warmed to body temperature, was administered intraperitoneally at a volume of 10 µl/g of body weight.

After anaesthesia, sub-cutaneous injection of buprenorphine 0.3 mg/ml (Buprecare, Axience, Patin, France) was administered at a dose of 0.08 mg/kg of body weight. Local anaesthesia using Laocaïne^®^ 2% (MSD Santé Animale, Beaucouzé, France) was administered sub-cutaneously at the surgical site at a dose of 2 mg/kg of body weight.

Artificial tears (Ocry-gel, TVM lab, Lempdes, France) were administered to both eyes. Loss of pedal withdrawal reflex of both hind paws was verified before incision, and was monitored during surgery. The cervical skin immediately below the ear was shaved and cleaned (Vétédine Solution, Vetoquinol, Magny-Vernois, France). Subcutanenous hydration was performed after the surgery and twice daily for 2 days.

### Behaviour evaluation

Mouse behaviour was assessed before and after surgery on day 1, day 4, day 7 and day 28. Normal locomotor mouse behaviour was assessed: ability to swim (over a 30-s-long period), to groom, to move in the cage and to reach for food or water. Vestibular postural and locomotor impaired behaviour was assessed: head tilt (inclination of the head towards the lesioned side), tumbling (mouse rolling around its longitudinal axis towards the lesioned side), twirl (while the mouse is being held by the tail) and circling (stereotyped movement in circles around the mouse’s hip). All eight items were quantified with a scale from 0 (normal behaviour, no deficit) to 3 (highest degree of abnormal behaviour), with a maximum deficit score of 24.

### Vestibular function exploration using video-oculography

To perform head restrained vestibular exploration, a head post was surgically implanted 2 weeks before the vestibular lesion. Head implant surgery and peri-operative care have been described previously [28, 29]. Briefly, under gas anaesthesia (isoflurane), a small custom-built head holder was cemented (C&B Metabond) to the skull just anterior to the lambda landmark (see França de Barros et al. 2019 for a video tutorial [30]).

Vestibular function was explored before vestibular surgery and after surgery at day 1, day 4, day 7 and long-term day 28. As reported previously, [9, 28, 29, 31] all eye movements recordings were made in the dark using an infrared video system (ETL-200, ISCAN, Burlington, MA, USA), recording pupil and corneal reflection (CR) position.

Eye movements were recorded using non-invasive video-oculography [32]. The experimental set-up, apparatus and methods of data acquisition were similar to those described previously [29, 33, 34]. Briefly, mice were head-fixed at a ~ 30° nose-down position to align the horizontal canals with the yaw plane [35]. Animals were placed in a custom-built Plexiglas tube secured on the superstructure of a vestibular stimulator. The VOR tests were performed in a temperature-controlled room (21 °C) with all sources of light turned off except for computer screens. The turntable was further enclosed in a box to isolate the animal from any remaining light, with a final luminance inside the box < 0.02 lx. Myosis was induced with topical 2% pilocarpine applied 10 min before experimentation. Recorded eye and head position signals were sampled at 1 kHz, digitally recorded (CED power1401 MkII) using Spike 2 software and later exported into the Matlab programming environment for off-line analysis (Matlab, The MathWorks).

i. Videonystagmography first recorded spontaneous eye movements without any vestibular stimulation, and number and direction of the nystagmus rate per minute were reported.
ii. Then, the angular horizontal vestibulo-ocular reflex (aVOR) was tested during horizontal sinusoidal rotation of the turntable (at 0.2; 0.5; 1 and 1.5 Hz; peak velocity 30°/s). Analysis was made on at least 10 cycles. Two parameters were extracted from the recordings: the gain and the phase. The gain was the ratio between the amplitude of the eye (response) and head (stimulus) rotations. Since the animal was head-fixed to the rotating table, head movements and table movements were identical. The phase was the temporal shift between the eye and table rotations, expressed in degrees as the ratio of the sinusoidal cycle (2 pi). Details for gain and phase calculation were reported in Carcaud et al. [33]. Values with VAF (variance-accounted-for) under 0.5 were discarded [28].
iii. The eye movements evoked by a specific stimulation of the otolith organs (maculo-ocular reflexes, MOR) were tested [36] using an off-vertical axis rotation (OVAR) as previously described [29]. Briefly, the axis of rotation was tilted from the vertical by 17°. Rotations were performed at a constant speed (50°/s) for at least 10 rotations both in clockwise (CW) and counterclockwise (CCW) directions. Due to the inertial nature of the angular movement detection, a rotation at constant speed elicits a combined canalar and otolithic response at the onset of movement, however, after a few seconds only the otolithic component remains [29, 37]. Since gravitational acceleration acts vertically, this stimulation is equivalent to a continuous rotation (at 0.14 Hz) around the mouse’s head of a 17° tilted constant linear acceleration stimulus (see Fig. 2b in Beraneck et al. [29]). For horizontal OVAR responses, quick-phases of reflexive eye movements were identified and not considered for analysis. During rotations, the velocity of horizontal slow phases is modulated (modulation, *μ*) around a constant bias (*β*). Both parameters (*μ* and *β*) were calculated from the sinusoidal fit of eye horizontal slow-phase velocity using the least-squares optimization of the equation:
  $$SP\left(t\right)=\beta +\mu \bullet \mathrm{sin} \left[2\pi \bullet {f}_{0}\bullet \left(t+{t}_{d}\right)\right]$$
  where SP(*t*) is the slow-phase velocity, *β* is the steady-state bias slow phase velocity, *μ* is the modulation of eye velocity, *f*_0_ is the frequency of table rotation, *t*_*d*_ is the dynamic lag time (in msec) of the eye movement with respect to the head movement. The bias (Maculo-ocular reflex Bias; MOR_*B*_) is reported here as the main index of the otolithic response [29, 36]. Notably, MOR requires normal otolith function but also an efficient central velocity storage network.
iv. Static ocular counterroll (OCR) was studied: vertical pupil position according to the head tilt angle was measured first with the mouse maintained at a 0° horizontal position. The platform was then tilted into different roll positions, at 10°, 20°, 30°, 40° and 50° alternatively to the right and to the left. The platform was rotated manually and slowly to limit semi-circular canal stimulation. Measurements were made in a static position during at least 15 s to identify the stable pupil position. The vertical eye angle was then calculated from the raw vertical CR and pupil position [38]. The slope of a linear regression of both variables (vertical eye angle and head tilt degree) was calculated, for ispilesional (− 50°–0°) and contralateral (0°–50°) sides.
v. Finally, angular velocity steps in the horizontal plane (hsteps) were performed at a speed of 50°/s. The horizontal slow phase velocity decay was fitted to an exponential curve (*f*(*x*) = a*exp(b*x)) and the time constant *τ* was then calculated as *τ* = − *1*/*b*. The time constant of the slow phase exponential velocity decay was calculated at the start and stop of CW and CCW rotations. CCW-start and CW-stop, CCW-stop and CW-start, were combined to assess left and right vestibular functions, respectively.

### Statistical analysis

Statistical analysis was made using XLstats (Addinsoft, New York, NY, USA). All data are reported as mean and standard deviation. Non-parametric means were compared with the Mann–Whitney test and proportions with the Fisher test. Repeated measures ANOVA was used, three-way to compare aVOR (lesion, time and frequency), two-way for ipsilateral and contralateral OVAR, angular velocity and static head-tilt stimulations and one-way for the behaviour score and nystagmus frequency count. Post-hoc comparisons were performed where appropriate using the Tukey HSD test. Values of *p* < 0.05 were considered significant.

## Surgical techniques

### Unilateral labyrinthectomy (UL) step-by-step surgical technique

**Fig. 1:**
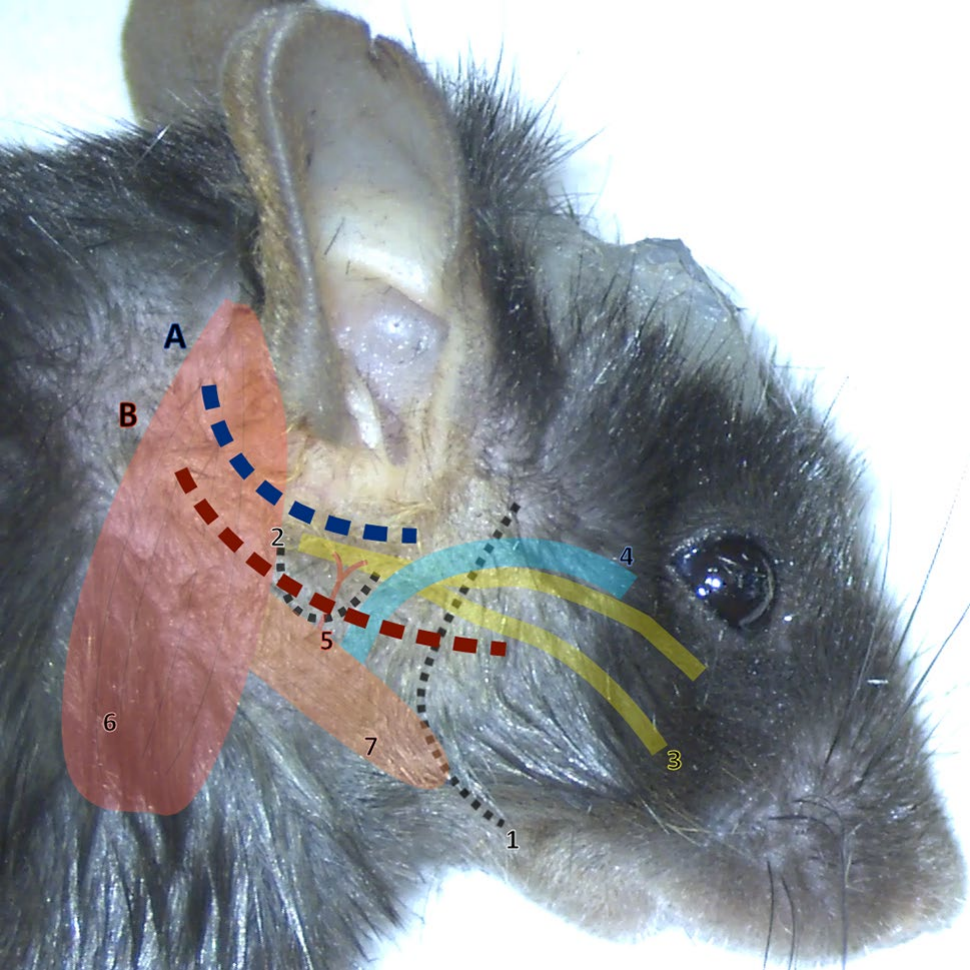
Incisions and main anatomical landmarks. **a** (In blue): posterior incision for labyrinthectomy, **b** (in red): inferior incision for neurectomy. 1: mandible; 2: bulla; 3: facial nerve; 4: superficial temporal vein; 5: temporal artery; 6: sternomastoid muscle; 7: digastric muscle. The superficial temporal vein is one of the two main bleeding risks with the stapedial artery (not shown on this image). The mandible, facial nerve, sternomastoid and digastric muscles are key landmarks to find the bulla. The head post is visible on the mouse’s skull, to be able to maintain the head in a fixed position during vestibular tests

The anaesthetised mouse was put in a side-lying position. A posterior incision of the skin following the external auditory canal anteriorly was performed (Fig. 1). The sub-cutaneous fat was incised immediately to find the cartilaginous external auditory canal, to dissect along with it in an avascular plane. Four retractors were positioned to open the cavity.

**Fig. 2:**
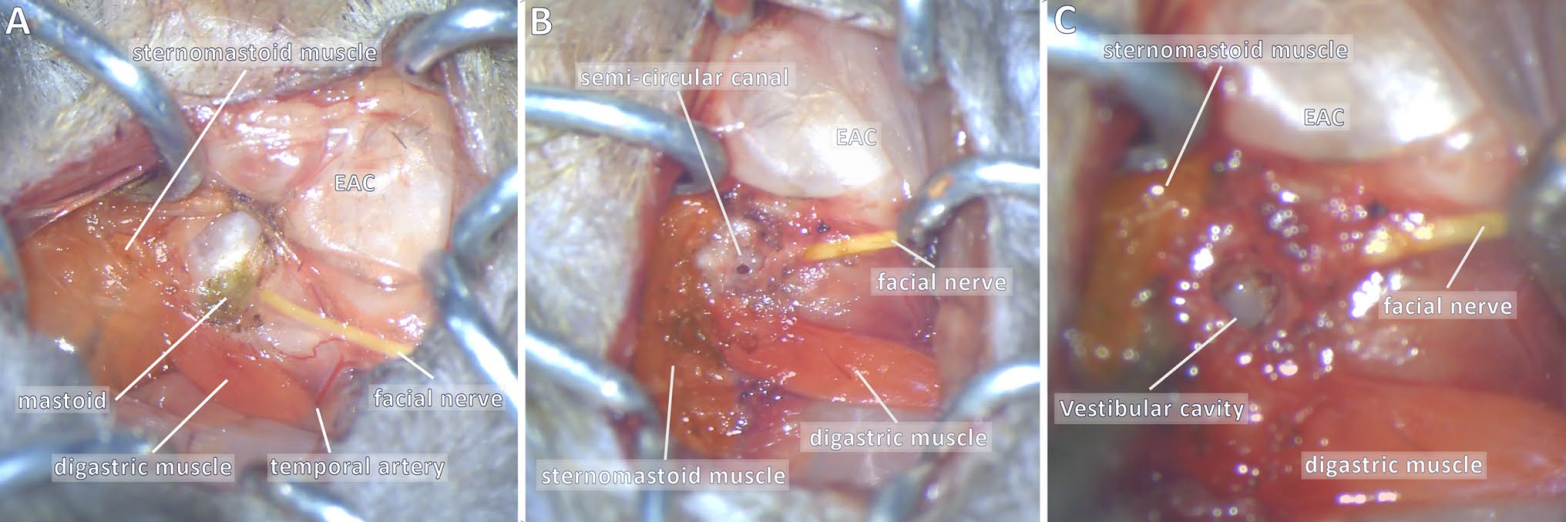
Unilateral labyrinthectomy: key steps. Key steps are shown: **a** exposure of the mastoid bone, **b** drilling of the mastoid, **c** opening of the vestibular cavity. The mastoid bone (shown in green) can be exposed after elevation of the sternomastoid muscle posteriorly. The following anatomical landmarks stand out: sternomastoid muscle posteriorly (orange), digastric muscle inferiorly (red) and external auditory canal (EAC) superiorly (white). The facial nerve is of particular interest as the mastoid is immediately posterior to its foramen, and may be used as a guide to find the mastoid in an anterio-posterior direction. Great care must be taken not do damage the stapedial artery which is immediately anterior to the cavity. Full size figures are available online (Supplementary Figs. 1, 2, 3)

The first visible anatomical landmarks were the facial nerve, external auditory canal and sternomastoid muscle. The facial nerve pointed towards the mastoid which was at this stage hidden beneath the sternomastoid muscle. Thus, the sternomastoid muscle was elevated from the bone, using a cautery to prevent bleeding. The muscle was then retracted posteriorly to expose the mastoid bone (Fig. 2a).

A 0.5 mm cutting burr was used to drill the mastoid, immediately posteriorly to the facial nerve foramen. The bone was not be drilled too posteriorly or superiorly as the cranial cavity would otherwise have been opened.

As the posterior semi-circular canal was opened (Fig. 2b), perilymphatic liquid oozed out and was suctioned as it can sometimes impair vision. The cavity was further opened with pointed instruments such as a hook or forceps. The drill would have been dangerous at this stage as it could have damaged the stapedial artery anteriorly or breached the cranial cavity posteriorly.

The contents (utricule, saccule and cupula) were suctioned until a clear and empty vestibular cavity was visible (Fig. 2c). The ganglion of Scarpa was left intact.

An absorbable gelatin compressed sponge was packed in the cavity and the skin was closed using simple interrupted absorbable 4–0 Vicryl (Ethicon, Somerville, NJ, USA).

### Unilateral vestibular neurectomy (UVN) step-by-step surgical technique

**Fig. 3:**
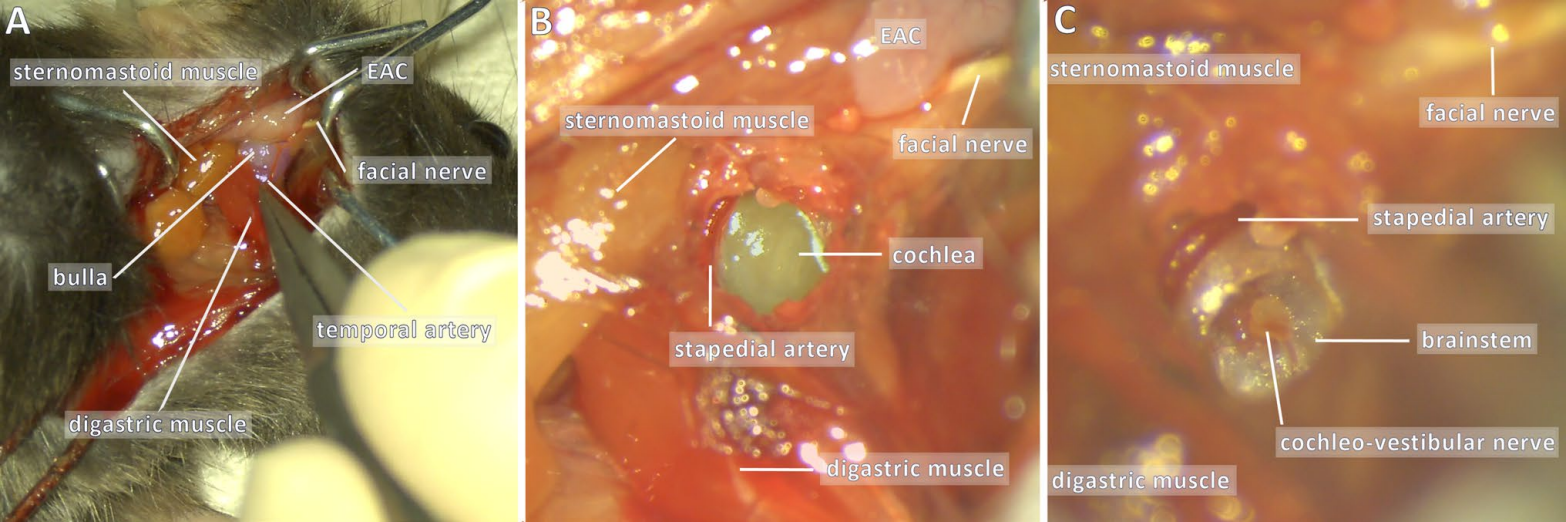
Unilateral vestibular neurectomy: key steps. Key steps are shown: **a** exposing the bulla, **b** opening the bulla and **c** exposition the vestibulo-cochlear nerve. An important anatomical landmark is the facial nerve, which can be followed backwards and which runs superiorly to the bulla (purple). Other anatomical landmarks include inferiorly the digastric muscle (red), posteriorly the sternomastoid muscle (orange) and the external auditory canal (EAC). The temporal artery also marks the bulla as it runs immediately superficially to the bulla, and must be cauterised to correctly drill the bulla. Full size figures are available online (Supplementary Figs. 4, 5, 6)

The technique was adapted from the previously published neurectomy procedure in the rat [21]. The anaesthetised mouse was put in a side-lying position. A ventral incision (Fig. 1) was performed, from the posterior limit of the external auditory canal to the mandible. After initial dissection of the subcutaneous fat, the first anatomical landmarks encountered were the jaw and masseter muscle, upon which lay the two branches of the facial nerve. Four retractors were put in place to open the surgical field. The superior branch of the nerve was parallel and close to the superficial temporal vein, which was retracted anteriorly to be protected. The superior retractor retracted the skin above the masseter, and the posterior retractor retracted the sternomastoid muscle (Fig. 3a).

The bulla was found by following the facial nerve posteriorly: the facial nerve passed horizontally between the insertion of the cartilaginous external auditory canal and the bulla.

The two major anatomical landmarks to locate the bulla were the digastric muscle inferiorly and the external auditory canal (and facial nerve) superiorly. The digastric muscle masked the posterior half of the bulla and was retracted. The bulla was also immediately beneath and slightly posterior to the Y-shaped temporal artery (Fig. 3a), which was cauterised to prevent bleeding. The bony surface of the bulla was exposed using a pointed instrument such as a hook or forceps.

The bulla was drilled using a 0.5 mm cutting burr, the two main vascular risks being the superficial temporal vein anteriorly and the stapedial artery in the middle ear cavity posteriorly. The bulla was cautiously opened until the stapedial artery was visualised (Fig. 3b).

Next, the cochlea was drilled with the same burr, carefully so as not to damage the stapedial artery. Perilymphatic fluid was suctioned to find the cochlear nerve. Residual bone was cleared using a hook and the cochlear nerve was then followed to reach the vestibulo-cochlear nerve and the brainstem. The VIIIth nerve was sectioned using a hook and the ganglion of Scarpa suctioned (Fig. 3c), great care was taken not to damage the brainstem underneath. The ganglion of Scarpa was visible as a bulge of the nerve, and the procedure was thus a neurectomy of the vestibular ganglion neurons (and not a neurotomy).

Gelfoam was packed in the cavity and the skin was closed using simple interrupted absorbable 4–0 Vicryl (Ethicon, Somerville, NJ, USA).

## Results

A total of seven mice underwent UL and ten underwent UVN. The survival rate was higher for UL (6/7 mice, 86%) than for UVN procedure (6/10 mice, 60%; *p* = 0.252). Concerning UL, one mouse did not wake up after anaesthesia, probably due to cardiogenic shock (no major bleeding during surgery). Concerning UVN, two mice died per-operatively, one immediately due to vascular damage and intense bleeding, and the other never woke up, with suspected continuous internal bleeding. The two other mice died post-operatively on day 3 and day 4, possibly due to neurological damage or cardiogenic shock. In none of the cases was there evidence for an infectious cause.

The mean age at surgery was 3.8 (range 2.5–6.3) and 4.5 (range 1.7–7.6) months, for UL and UVN, respectively. Mean operative time (from incision to closure) was 16.3 min (range 11–27) for UL and 20.5 min (range 18–25) for UVN, *p* = 0.199. 50% of UL and 100% of UVN had post-operative total right facial paralysis, although none of the nerves were deliberately cut. Partial recuperation at 1 month was observed.

### UL and UVN clinical follow-up

**Fig. 4:**
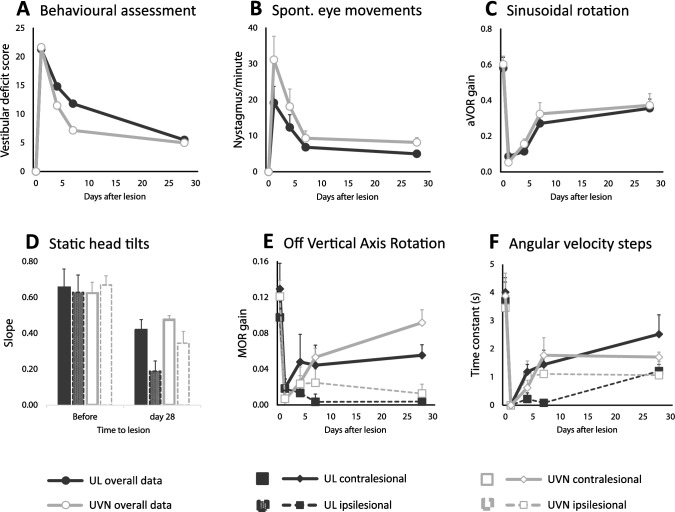
Vestibular function and behaviour of unilateral vestibular neurectomy vs labyrinthectomy. All graphs show the five time points, before surgical lesion, and during follow-up at day 1, day 4, day 7 and day 28 after lesion. Points represented are mean value with SEM (standard error of the mean). Statistical comparisons are reported in Tables 1, 2 and 3. **a** Behavioural assessment: vestibular deficit score is represented (maximum score of 24). **b** Spontaneous eye movements: spontaneous recording of the pupil in the dark, reporting the number of nystagmus in 1 min. Fast-beating component was always directed towards the contralateral side of the lesion (left side). **c** Sinusoidal rotation: results showing aVOR gain for a 0.5 Hz and 30°/s stimulation, demonstrating horizontal semi-circular canal function. **d** Static ocular counterroll: the slope of a linear equation is reported, of static lateral tilt degree and vertical position of the pupil, corresponding to utricular function. Only the reliable post-lesion results are shown at day 28. **e** Off Vertical Axis Rotation: clockwise and counter-clockwise rotations in yaw plane of a 17° tilted platform at a rotation of speed of 50°/s. MOR gain is reported, which corresponds to a complex integration of otolithic function and velocity storage. **f** Angular velocity steps: horizontal steps including three full rotations in yaw plane at 50°/s were analysed for exponential velocity decay time constant (at start and stop of clockwise and counter-clockwise rotations). Ipsilesional stimulation (right side) corresponds to CW-start and CCW-stop, contralesional stimulation corresponds to CCW-start and CW-stop stimulations

Initial clinical follow-up was identical in both UL and UVN mice. Before the animal regained full consciousness after anaesthesia, the following signs demonstrated a successful procedure: head tilted > 45° and body leaning towards the operated side, also the tail 90° bent towards the lesioned side. In the hours after surgery, the main behaviour indicating vestibular impairment was intense and frequent spontaneous tumbling (towards the operated side). The behavioural score was not statistically different at day 1 or after 1 month (Table 1), even though UL mice tended to recover more quickly than UVN mice (Fig. 4a). At day 4, all mice from both groups were able to reach for food and drink autonomously. The first vestibular symptom to disappear was tumbling (0/6 UL mice and 1/6 UVN mice at day 4). At 1 month, circling behaviour had completely disappeared in both groups, while all mice still had a noticeable head tilt (< 45°) and twirled when held by the tail towards the side of the lesion. None were able to swim or float (all presented with underwater tumbling).

**Table 1:** Behavioural score and nystagmus count

Lesion	UL	UVN	*p*
Behavioural score			
Before	0	0	–
Day 1	21.7 ± 1.2	21.3 ± 1.4	*0.522*
Day 4	**11.5 ± 1.4**	**14.8 ± 0.8**	**< 0.001**
Day 7	**7.2 ± 0.4**	**11.8 ± 1.2**	**< 0.001**
Day 28	5.0 ± 0.6	5.5 ± 0.5	*0.339*
Nystagmus count (beats per minute)			
Before	0	0	–
Day 1	**19 ± 11**	**31 ± 16**	***0.005***
Day 4	12 ± 9	18 ± 12	*0.161*
Day 7	7 ± 2	9 ± 5	*0.544*
Day 28	5 ± 1	8 ± 3	*0.746*

All values are represented as mean ± SD (standard deviation). Statistically significant values are in bold. Nystagmus count was calculated during spontaneous eye movement in the dark. Behavioural score and nystagmus count values were each compared between UL and UVN mice populations, using a two-way repeated ANOVA and post-hoc Tukey test, both model characteristics were (40, *p* < 0.0001)

*UL *unilateral labyrinthectomy, *UVN *unilateral vestibular neurectomy

### UL and UVN functional follow-up

Recordings of spontaneous eye movements always showed a nystagmus with the rapid beat towards the opposite side of the lesion (no nystagmus reported before surgery). Beats/min were maximal at day 1 (statistically higher after UVN lesions) and progressively decreased (Fig. 4b) for both UL and UVN lesions (Table 1). Sinusoidal rotations at 30°/s were overall not statistically different between UL and UVN lesions concerning gain or phase (Table 2). The gain values were at their lowest at day 1 with an improvement during the first week followed by a stabilisation (Fig. 4c). The gain was lower at day 28 compared to prelesion, for UL (*p* = 0.095, *p* < 0.001, *p* = 0.001) and for UVN (*p* = 0.011, *p* < 0.001, *p* < 0.001), at 0.2 Hz, 0.5 Hz and 1 Hz, respectively. Phase values were not interpretable at day 1 and day 4 with VAF < 0.5, and thus were excluded from the analysis (Table 2).

**Table 2:** Canalar function (sinusoidal rotation at 30°/s): aVOR gain and phase

Frequency	0.2 Hz			0.5 Hz			1 Hz		
Lesion	UL	UVN	*p*	UL	UVN	*p*	UL	UVN	*p*
aVOR gain									
Before	0.25 ± 0.09	0.35 ± 0.12	*0.107*	0.58 ± 0.15	0.60 ± 0.11	*0.712*	0.66 ± 0.17	0.74 ± 0.20	*0.192*
Day 1	0.04 ± 0.04	0.03 ± 0.02	*0.865*	0.09 ± 0.05	0.05 ± 0.03	*0.570*	0.17 ± 0.11	0.10 ± 0.07	*0.294*
Day 4	0.05 ± 0.05	0.07 ± 0.06	*0.887*	0.12 ± 0.07	0.16 ± 0.07	*0.478*	**0.16 ± 0.07**	**0.29 ± 0.09**	***0.030***
Day 7	0.12 ± 0.08	0.16 ± 0.12	*0.514*	0.27 ± 0.12	0.33 ± 0.16	*0.364*	0.42 ± 0.21	0.48 ± 0.18	*0.335*
Day 28	0.16 ± 0.06	0.20 ± 0.07	*0.478*	0.36 ± 0.13	0.37 ± 0.16	*0.776*	0.47 ± 0.16	0.49 ± 0.13	*0.820*
aVOR phase									
Before	29.3 ± 8.3	24.5 ± 10.6	*0.649*	9.9 ± 8.7	10.2 ± 3.9	*0.982*	− 2.2 ± 4.3	− 0.2 ± 3.6	*0.855*
Day 1*	–	–	–	–	–	–	–	–	–
Day 4*	–	–	–	–	–	–	–	–	–
Day 7	39.6 ± 9.4	31.8 ± 22.0	*0.457*	39.2 ± 25.6	23.1 ± 13.2	*0.126*	23.2 ± 14.6	16.0 ± 25.0	*0.492*
Day 28	43.6 ± 6.3	26.3 ± 41.9	*0.102*	27.9 ± 6.1	25.0 ± 11.4	*0.393*	5.8 ± 7.5	6.8 ± 4.4	*0.925*

All values are represented as mean ± SD (standard deviation). Statistically significant values are in bold. Gain and phase values were each compared between UL and UVN mice populations, using a three-way repeated ANOVA and post-hoc Tukey test, gain model (150, *p* < 0.0001) and Phase model (90, *p* < 0.0001)

*aVOR* angular vestibular-ocular reflex, *UL* unilateral labyrinthectomy, *UVN* unilateral vestibular neurectomy

*Phase was not measurable at day 1 or day 4

Static ocular counterroll as well as responses to off-vertical axis rotation (OVAR) and angular velocity steps were analysed during rotation towards the ipsilesional and contralesional sides (Table 3). Briefly, static ocular counterroll was reduced at day 28 compared to prelesion (Fig. 4d), for tilts towards the ipsi- and contralesional side in UL mice (*p* < 0.001, *p* = 0.027) and UVN mice (*p* = 0.003, *p* = 0.159). At day 28, concerning UL, the ocular counterroll measured during inclination towards the contralesional side was statistically greater than when measured during inclination towards the ipsilesional side (0.42 vs 0.19, *p* = 0.033). This difference in the ocular counterroll observed during rotation toward the ipsilateral or contralateral side did not reach significance for UVN (0.47 vs 0.34, *p* = 0.220). We noted an important skew deviation (with the contralesional eye moving upwards) at day 1 and day 4 in both UVN and UL.

**Table 3:** Velocity storage and otolithic function

Stimulation	Ipsilesional			Contralesional		
Lesion	UL	UVN	*p*	UL	UVN	*p*
Static head tilt, slope*						
Before	0.63 ± 0.24	0.67 ± 0.12	*0.701*	0.65 ± 0.25	0.62 ± 0.14	*0.781*
Day 1	0.16 ± 0.26	0.37 ± 0.27	*0.053*	0.57 ± 0.20	0.42 ± 0.28	*0.169*
Day 4	0.15 ± 0.11	0.28 ± 0.14	*0.221*	0.31 ± 0.28	0.34 ± 0.15	*0.814*
Day 7	0.19 ± 0.07	0.22 ± 0.12	*0.811*	0.41 ± 0.23	0.25 ± 0.18	*0.144*
Day 28	0.19 ± 0.14	0.34 ± 0.16	*0.493*	0.42 ± 0.14	0.47 ± 0.06	*0.143*
Off-vertical-axis rotation, MOR gain						
Before	0.10 ± 0.07	0.12 ± 0.04	*0.312*	0.13 ± 0.07	0.10 ± 0.05	*0.197*
Day 1	0.02 ± 0.02	0.01 ± 0.01	*0.274*	0.02 ± 0.03	0.01 ± 0.02	*0.292*
Day 4	0.01 ± 0.03	0.02 ± 0.02	*0.110*	**0.05 ± 0.08**	**0.03 ± 0.04**	***0.002***
Day 7	0.00 ± 0.02	0.02 ± 0.04	*0.222*	0.04 ± 0.05	0.05 ± 0.02	*0.702*
Day 28	0.00 ± 0.01	0.01 ± 0.03	*0.694*	0.06 ± 0.03	0.09 ± 0.04	*0.114*
Angular velocity steps, time constant (seconds)						
Before	3.69 ± 1.67	3.46 ± 1.27	*0.690*	4.01 ± 1.26	3.88 ± 1.97	*0.826*
Day 1	0.00 ± 0.00	0.00 ± 0.00	*1*	0.00 ± 0.00	0.00 ± 0.00	*1*
Day 4	0.22 ± 0.54	1.02 ± 1.35	*0.176*	1.19 ± 0.63	0.62 ± 0.91	*0.336*
Day 7	0.08 ± 0.15	1.11 ± 1.62	*0.084*	1.44 ± 1.24	1.77 ± 1.53	*0.576*
Day 28	1.21 ± 0.58	1.07 ± 1.20	*0.807*	2.52 ± 1.68	1.71 ± 0.43	*0.170*

All values are represented as mean ± SD (standard deviation). Statistically significant values are in bold. Static head tilt, Off-vertical-axis rotation (OVAR) and angular velocity steps values were each compared between UL and UVN mice populations, using a three-way repeated ANOVA and post-hoc Tukey test, all three model characteristics were (100, *p* < 0.0001)

*UL* unilateral labyrinthectomy, *UVN* unilateral vestibular neurectomy

*Slope of a linear regression of both variables (vertical eye angle and head tilt degree)

Concerning OVAR, MOR gain was lowest at day 1, and remained statistically lower at day 28 compared to prelesion (Fig. 4e), for rotation towards the ipsilateral side but not towards the contralesional side for UL (*p* < 0.001, *p* = 0.746) and UVN (*p* < 0.001, *p* = 0.729). At day 28, gain for rotation towards the contralesional side was statistically higher than during rotations towards the ipsilesional side for UL (0.06 vs 0.00, *p* = 0.011) and for UVN (0.09 vs 0.01, *p* < 0.001). There was no statistically significant difference between UVN and UL mice (except at day 4 concerning rotation towards the contralesional side).

Lastly, angular velocity steps showed time constants of the velocity decay lowest at day 1, which remained significantly lower at day 28 compared to prelesion (Fig. 4e), for both rotations towards ipsi- and contralesional sides in UL (*p* < 0.001, *p* = 0.013) and in UVN mice (*p* < 0.001, *p* < 0.001). At day 28, in UL mice, the contralesional time constant was significantly higher than the ipsilesional time constant (2.51 vs 1.21, *p* = 0.028) but not for UVN (1.71 vs 1.07, *p* = 0.277). There was no statistically significant difference between UVN and UL mice.

## Discussion

Two different surgical strategies to achieve complete vestibular lesions may be used in the mouse, UL (preganglionic lesion) which destroys the peripheral labyrinthine organs and UVN (postganglionic lesion) which destroys the first order vestibular neurons in the ganglion of Scarpa (and is thus a neurectomy of the VIIIth nerve). A summary of the differences is presented in Table 4.

**Table 4:** Summary of differences between unilateral labyrinthectomy and unilateral vestibular neurectomy

Lesion	UL	UVN
Surgical procedure length	Shortest (16 min)*	Longest (21 min)*
Mouse survival rate	Satisfactory (86%)*	Poor (60%)*
Cochlea	Intact	Destroyed
Vestibular labyrinth	Destroyed	Intact
Ganglion of Scarpa	Intact	Destroyed (suctioned)
Type of lesion	Preganglionic lesion	Postganglionic lesion
Degeneration of afferent fibres	No	Yes
Behavioural score	Shorter recovery	Longer recovery
Vestibular function	Similarly impaired	Similarly impaired

*No statistically significant difference

In both cases, surgical landmarks are key, mainly the facial nerve, the external auditory canal and sternomastoid and digastric muscles, to swiftly find the mastoid (UL) or bulla (UVN). It is also important to operate with a good quality microscope (especially for the neurectomy step) and with an electric cautery. Ketamine-xylazine general anaesthesia was preferred as isoflurane is known to induce vasodilation [39]. In addition, in the mouse, manipulation of the head during surgery may be difficult with a facial mask or intubation necessary for isoflurane. Post-operative care is paramount in both surgeries but especially UVN, to prevent dehydration (as mice are unable to drink on their own due to tumbling during the first 48 h) but also to prevent ipsilateral keratitis if the facial nerve was damaged. The auditory function cannot be preserved due to the surgical destruction of the labyrinth, especially in the UVN procedure and its transcochlear approach (thus was not tested in this study).

UVN surgery seemed more severe and aggressive than UL: the behavioural score was significantly higher at day 4 and 7 and the number of nystagmus beats per minute was higher; however, vestibular compensation followed a comparable time course, both after 1 week and at 1 month.

Thus, for the same overall vestibular deficit, in our hands UL is preferred, as the procedure is shorter and bleeding and neurological per-operative risks are reduced with higher survival rates compared to UVN.

The destruction of the vestibular neurons in UVN (postganglionic lesion) induces Wallerian degeneration of the entire vestibular nerve and an inflammatory response reaching the vestibular nuclei [21, 40, 41]. Indeed degeneration of afferent fibres seems to take place after post- but not preganglionic lesions [42]. Such intense inflammation may be key in certain research protocols, in which case the choice of UVN over UL is justified. This has been shown in research on central vestibular neurogenesis, where the neurogenic potential has been identified after UVN but not after UL or pharmacological vestibular lesions [24, 43–45].

Although this was not the main objective of the study, the small number of mice limits the fine interpretation of functional results especially concerning ipsilesional and contralesional data. Some hypothesis may be drawn from our statistics, as contralesional stimulation partially recuperated in most cases at 1 month, in line with previous reports [14]. Concerning overall vestibular function after a unilateral vestibular lesion, this study confirms other papers showing a fast functional compensation period which corresponds to rapid behavioural improvement during the first post-operative week (especially during the first 3 days), which tends to stabilise with a prolonged hypofunction and near-normal behaviour at 1 month, except swimming which did not recuperate [11, 28]. After initial poor bilateral MOR gain, gain improved in both UVN and UL mice at 1 month when the contralesional side was stimulated. The mechanism is unclear (it may reflect asymmetric otolith input to the central nervous system or interactions between semi-circular canal-ocular and otolith-ocular reflexes) but it has been reported previously that the bias component remains either small or in an inappropriate direction during OVAR ipsilesional stimulation [46, 47]. Skew deviation is a well described vertical contralesional pupil elevation after peripheral vestibular lesions, whether of surgical, pharmacological or pathological origin [48, 49]. Regarding vertical eye position during static head tilt, skew deviation limited the capacity to measure the response, especially during tilts towards the contralesional side from day 1 to 4. Thus, the impairment was greatest at day 4 (UL) or 7 (UVN), and then partially recuperated. Lastly, concerning the canalar time constant measured during horizontal steps, there was no statistically significant difference between UL and UVN mice. However, UL contralesional values almost returned to perilesional values and were statistically different from ipsilesional values; whereas in UVN, ipsi- and contralesional values were not statistically different at 1 month. It is possible that the Wallerian degeneration of the nerve in UVN, inducing central vestibular nuclei inflammation, may impact velocity storage bilaterally, while UL remaining peripheral inputs allows for restoration of the response during rotation towards the contralesional side at 1 month. These hypotheses should be verified by dedicated studies.

## Conclusion

UL and UVN are two reproducible surgical techniques to induce definite and total unilateral vestibular lesions. In both cases, surgical landmarks are key to quickly identify the vestibular system and protect blood vessels and the cranial cavity. These are mainly the facial nerve, the external auditory canal and the sternomastoid and digastric muscles. UVN and UL induce similar behavioural and functional vestibular deficits during a 1-month follow-up period. Thus, UL should be preferred in most cases as the procedure seems technically safer and quicker, with a higher survival rate. UVN can, however, be required in certain research protocols as the ganglion of Scarpa is destroyed, and Wallerian degeneration may spread inflammation to the central vestibular nuclei.

## Electronic supplementary material

Below is the link to the electronic supplementary material.

Supplementary file1 (XLSX 267 kb)

Supplementary file2. Supplementary Fig 1. Unilateral labyrinthectomy: exposure of the mastoid bone. The mastoid bone (shown in green) can be exposed after the elevation of the sternomastoid muscle posteriorly. The following landmarks stand out: sternomastoid muscle posteriorly (orange), digastric muscle inferiorly (red) and external auditory canal (EAC) superiorly (white). The facial nerve is of particular interest as the mastoid is immediately posterior to its foramen, and may be used as a guide to find the mastoid in an anterio-posterior direction (TIF 2212 kb)

Supplementary file3. Supplementary Fig 2. Unilateral labyrinthectomy: opening the vestibule. The mastoid is drilled immediately posterior to the facial nerve (if the bone is drilled too posteriorly or too superiorly, the cranium may be opened). The posterior semi-circular canal is the first to be opened which is confirmed with perilymphatic liquid (see video). The same landmarks are found as in Figure 2: sternomastoid and digastric muscles, facial nerve, external auditory canal (EAC) (TIF 2025 kb)

Supplementary file4. Supplementary Fig 3. Unilateral labyrinthectomy: opening the vestibular cavity. The vestibular cavity is drilled and suctioned to destroy and remove the ampulas, utricle and saccule. Great care must be taken not do damage the stapedial artery which is immediately anterior to the cavity. The same anatomical landmarks can still be seen: sternomastoid and digastric muscles, facial nerve, external auditory canal (EAC) (TIF 2004 kb)

Supplementary file5. Supplementary Fig 4. Unilateral vestibular neurectomy: exposing the bulla. The first step is to expose the bulla (in purple), which is immediately below the external auditory canal (EAC). Another clear anatomical landmark is the facial nerve, which can be followed backwards and which runs superiorly to the bulla. Other anatomical landmarks include inferiorly the digastric muscle (red) and posteriorly the sternomastoid muscle (orange). The temporal artery also marks the bulla as it runs immediately superficially to the bulla, and must be later cauterised to correctly drill the bulla (TIF 5510 kb)

Supplementary file6. Supplementary Fig 5. Unilateral vestibular neurectomy: opening the bulla. The bulla must be drilled taking great care not to damage the superficial temporal vein (not visible here) anteriorly, and the stapedial artery posteriorly which is in the middle ear. The cochlea’s spiral shape can be seen through the opening. Other landmarks are visible: sternomastoid and digastric muscles, facial nerve, external auditory canal (EAC) (TIF 3044 kb)

Supplementary file7. Supplementary Fig 6. Unilateral vestibular neurectomy: vestibulo-cochlear nerve. The cochlea is then drilled taking care not to damage the stapedial artery posteriorly. The cochlear nerve (not shown) is followed until the vestibulo-cochlear nerve is visible exiting the brainstem, at which point it is cut and suctioned with the ganglion of Scarpa (bulge in the nerve). Great care must be taken not to damage the brainstem and keep the meninges intact. Other landmarks are visible: sternomastoid and digastric muscles, facial nerve, external auditory canal (EAC) (TIF 2897 kb)

Supplementary file8. Video: an educational surgical video of both unilateral labyrinthectomy and unilateral vestibular neurectomy is included (MP4 204145 kb)
