# Supplementary figures and images for: Surgical techniques and functional evaluation for vestibular lesions in the mouse: unilateral labyrinthectomy (UL) and unilateral vestibular neurectomy (UVN)
Source: J Neurol. 2020 Jun 17;267(Suppl 1):51–61. doi: 10.1007/s00415-020-09960-8 (PMC7718198; doi:10.1007/s00415-020-09960-8)

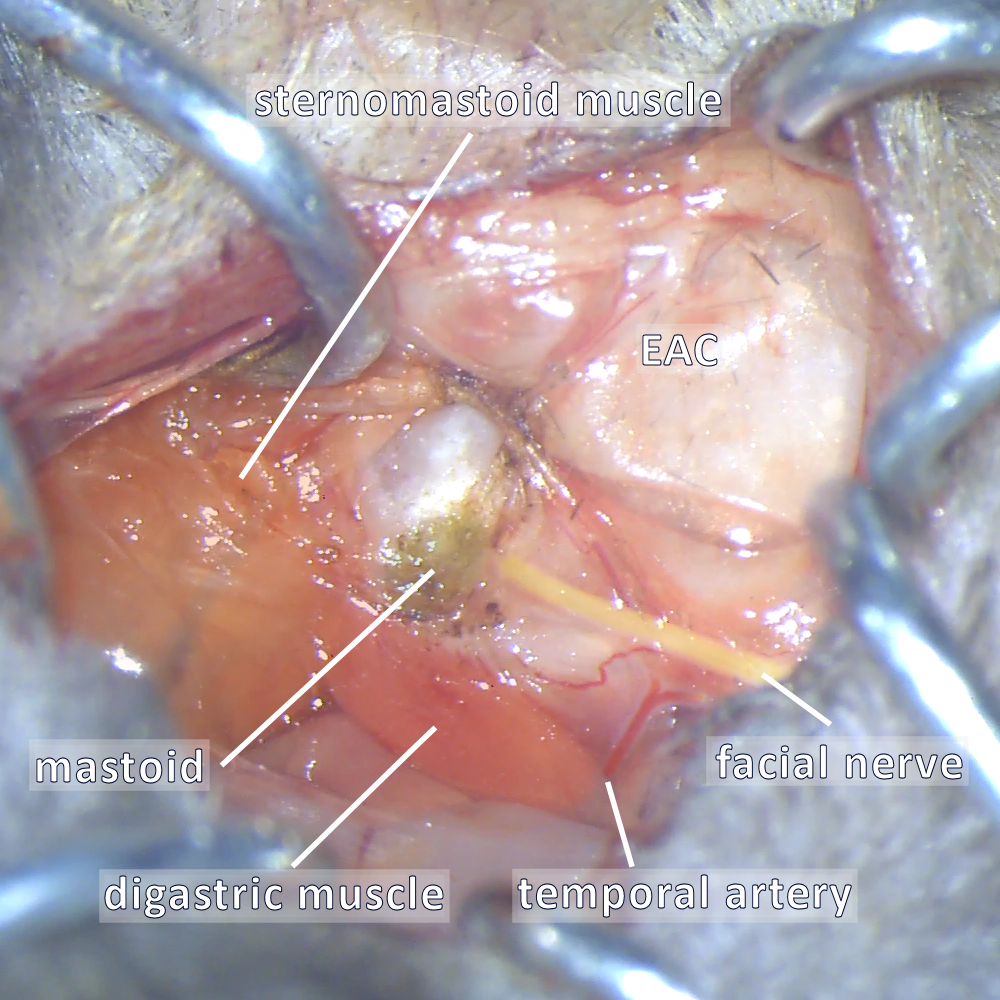

Supplement: Supplementary file 2 — Supplementary file2. Supplementary Fig 1. Unilateral labyrinthectomy: exposure of the mastoid bone. The mastoid bone (shown in green) can be exposed after the elevation of the sternomastoid muscle posteriorly. The following landmarks stand out: sternomastoid muscle posteriorly (orange), digastric muscle inferiorly (red) and external auditory canal (EAC) superiorly (white). The facial nerve is of particular interest as the mastoid is immediately posterior to its foramen, and may be used as a guide to find the mastoid in an anterio-posterior direction (TIF 2212 kb) [file 415_2020_9960_MOESM2_ESM.tif]

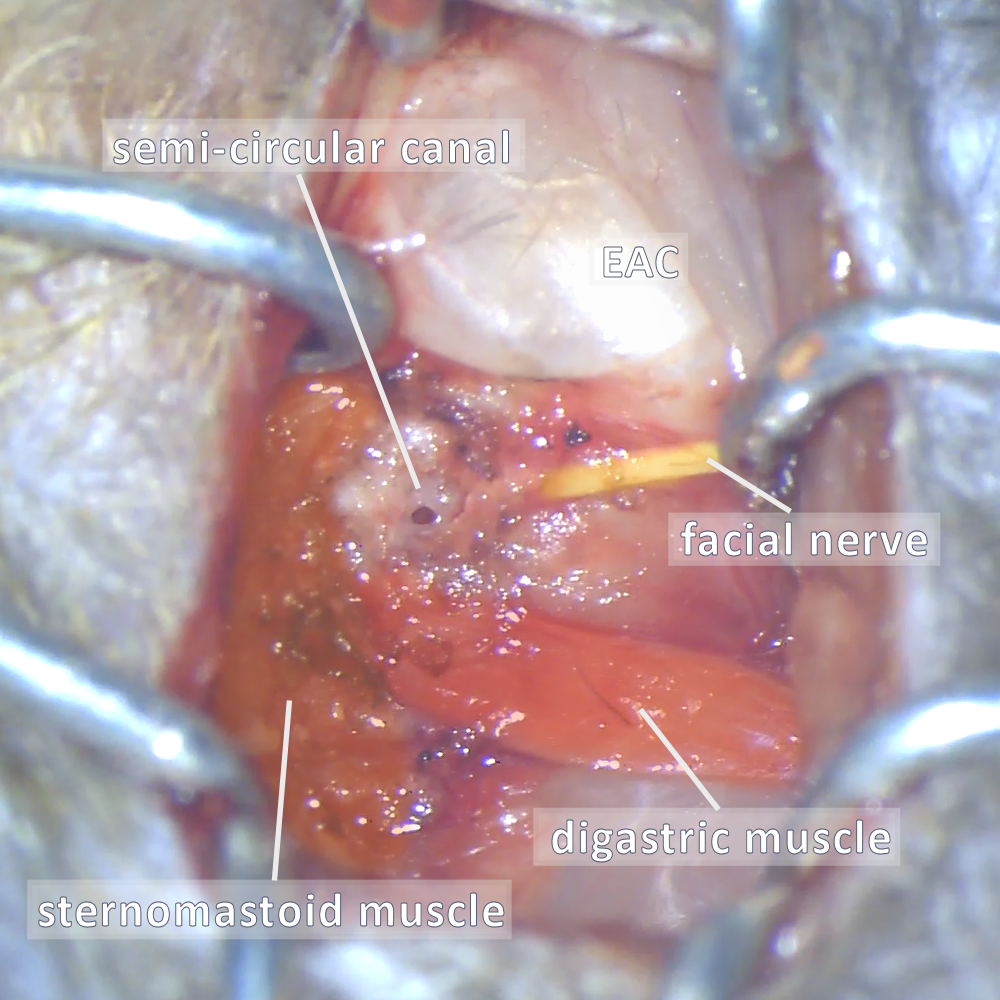

Supplement: Supplementary file 3 — Supplementary file3. Supplementary Fig 2. Unilateral labyrinthectomy: opening the vestibule. The mastoid is drilled immediately posterior to the facial nerve (if the bone is drilled too posteriorly or too superiorly, the cranium may be opened). The posterior semi-circular canal is the first to be opened which is confirmed with perilymphatic liquid (see video). The same landmarks are found as in Figure 2: sternomastoid and digastric muscles, facial nerve, external auditory canal (EAC) (TIF 2025 kb) [file 415_2020_9960_MOESM3_ESM.tif]

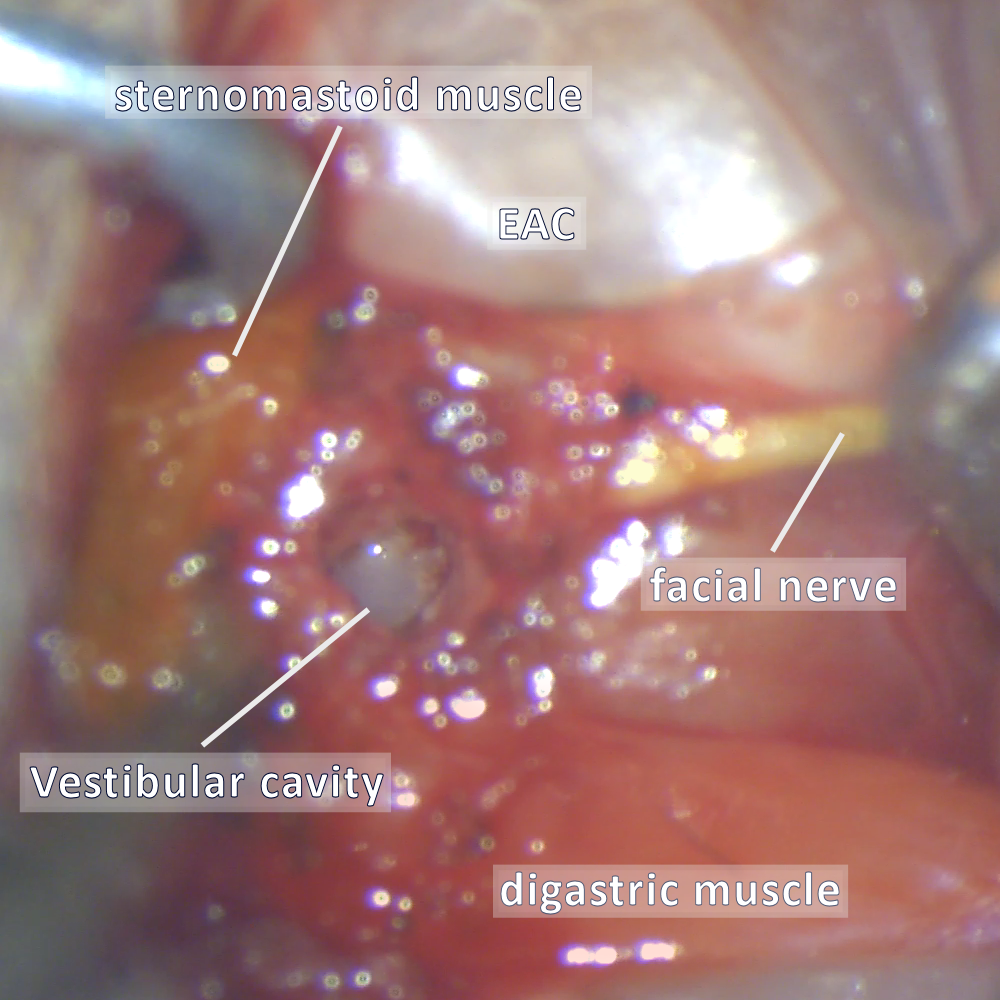

Supplement: Supplementary file 4 — Supplementary file4. Supplementary Fig 3. Unilateral labyrinthectomy: opening the vestibular cavity. The vestibular cavity is drilled and suctioned to destroy and remove the ampulas, utricle and saccule. Great care must be taken not do damage the stapedial artery which is immediately anterior to the cavity. The same anatomical landmarks can still be seen: sternomastoid and digastric muscles, facial nerve, external auditory canal (EAC) (TIF 2004 kb) [file 415_2020_9960_MOESM4_ESM.tif]

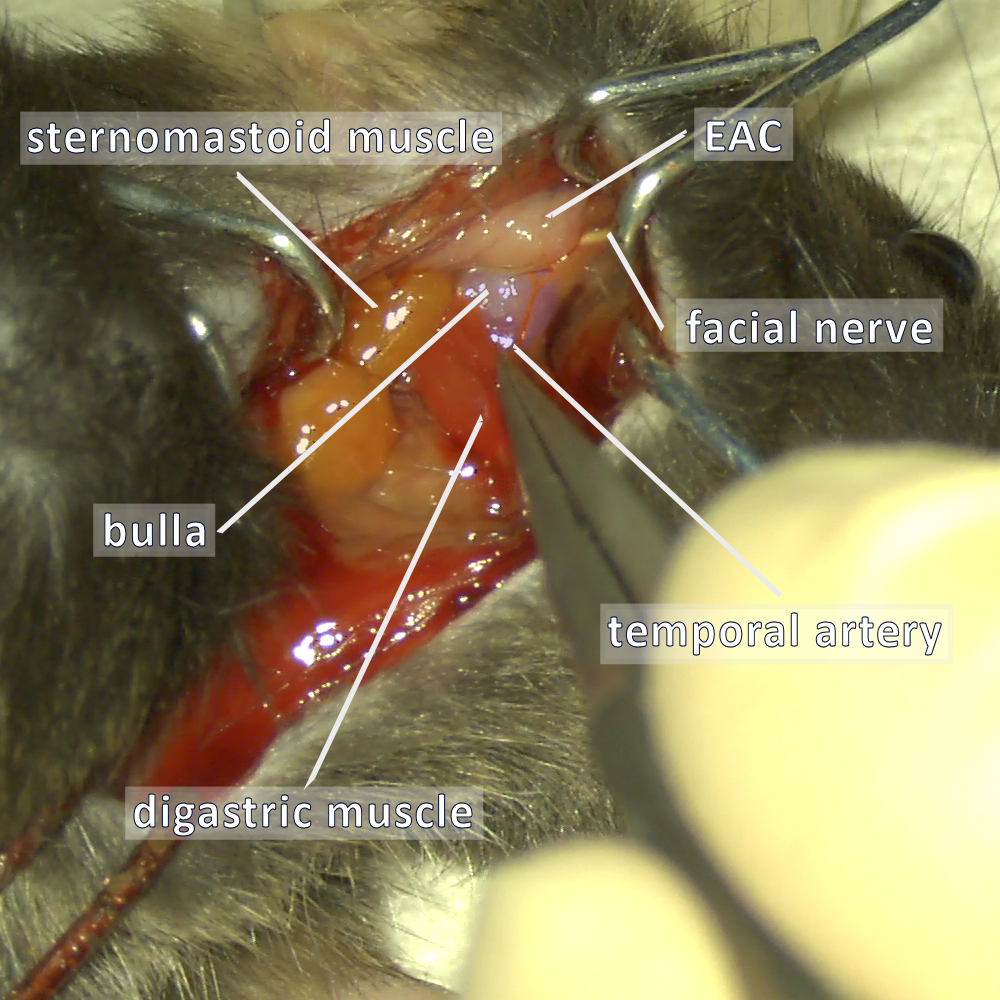

Supplement: Supplementary file 5 — Supplementary file5. Supplementary Fig 4. Unilateral vestibular neurectomy: exposing the bulla. The first step is to expose the bulla (in purple), which is immediately below the external auditory canal (EAC). Another clear anatomical landmark is the facial nerve, which can be followed backwards and which runs superiorly to the bulla. Other anatomical landmarks include inferiorly the digastric muscle (red) and posteriorly the sternomastoid muscle (orange). The temporal artery also marks the bulla as it runs immediately superficially to the bulla, and must be later cauterised to correctly drill the bulla (TIF 5510 kb) [file 415_2020_9960_MOESM5_ESM.tif]

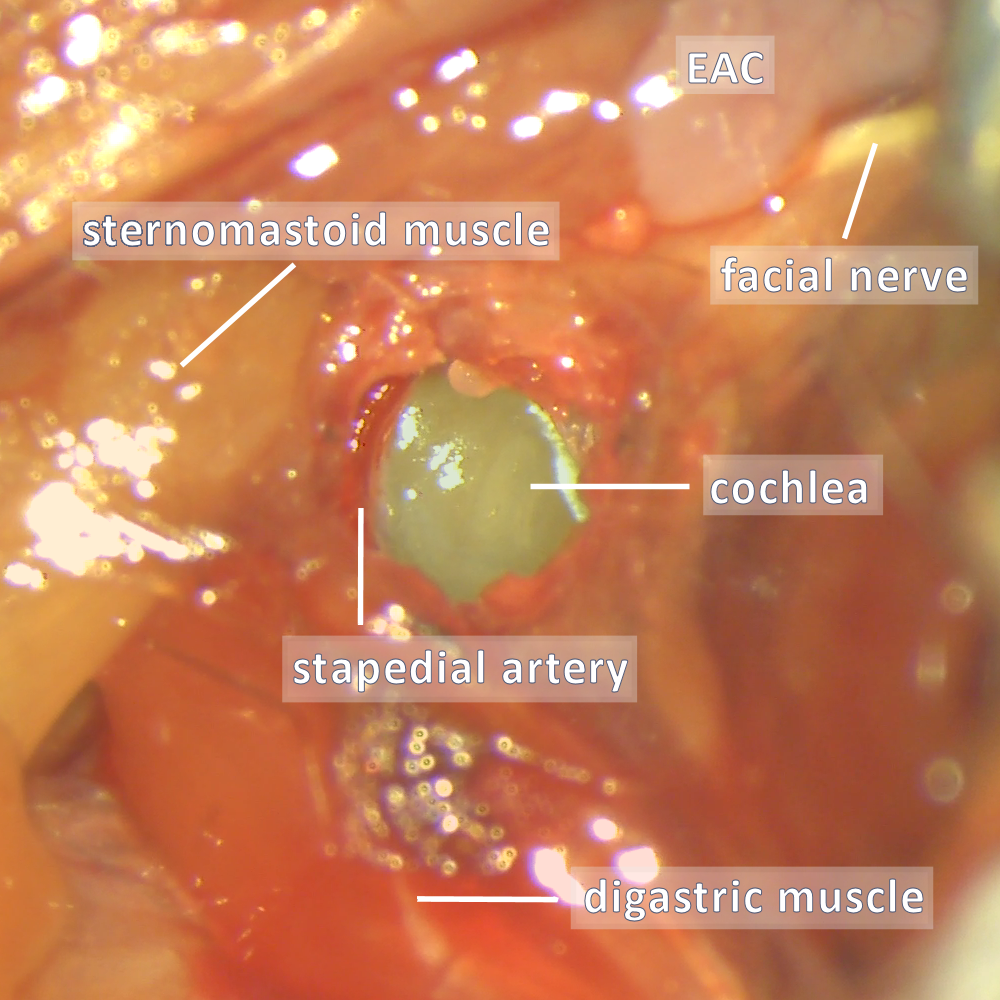

Supplement: Supplementary file 6 — Supplementary file6. Supplementary Fig 5. Unilateral vestibular neurectomy: opening the bulla. The bulla must be drilled taking great care not to damage the superficial temporal vein (not visible here) anteriorly, and the stapedial artery posteriorly which is in the middle ear. The cochlea’s spiral shape can be seen through the opening. Other landmarks are visible: sternomastoid and digastric muscles, facial nerve, external auditory canal (EAC) (TIF 3044 kb) [file 415_2020_9960_MOESM6_ESM.tif]

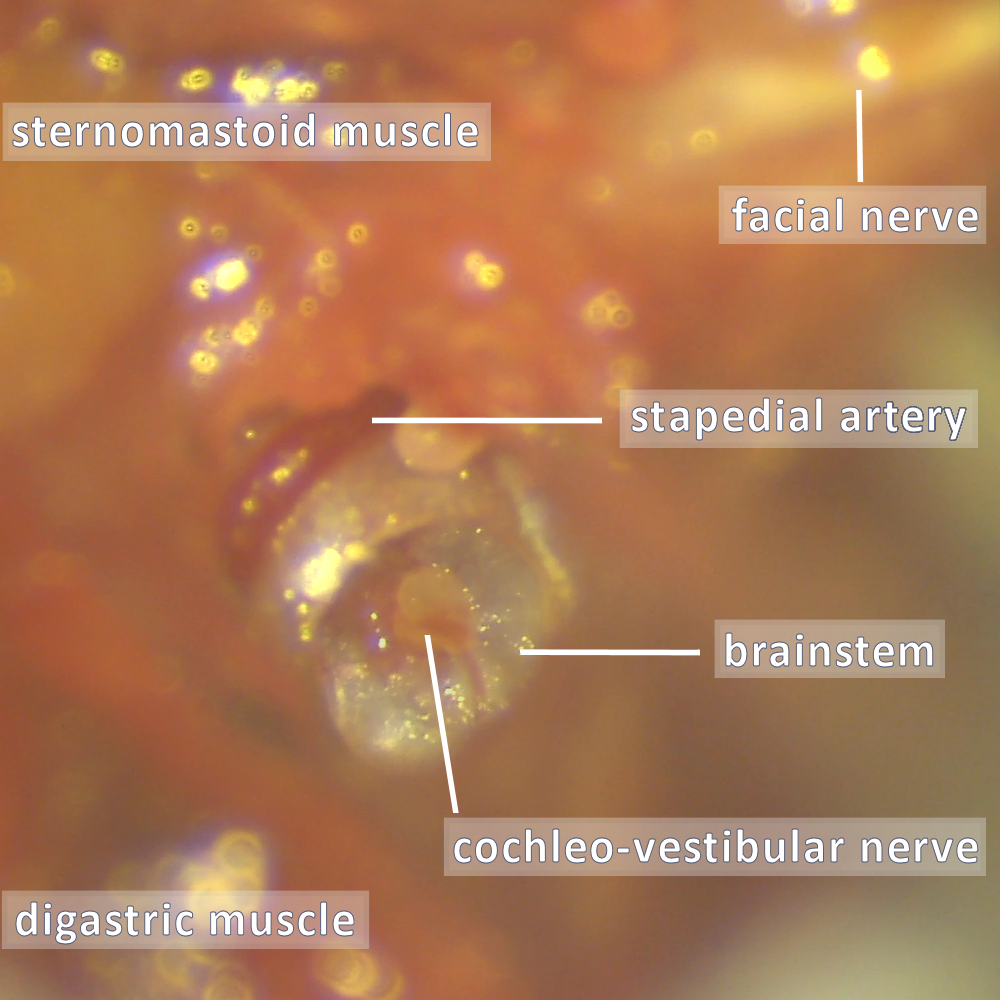

Supplement: Supplementary file 7 — Supplementary file7. Supplementary Fig 6. Unilateral vestibular neurectomy: vestibulo-cochlear nerve. The cochlea is then drilled taking care not to damage the stapedial artery posteriorly. The cochlear nerve (not shown) is followed until the vestibulo-cochlear nerve is visible exiting the brainstem, at which point it is cut and suctioned with the ganglion of Scarpa (bulge in the nerve). Great care must be taken not to damage the brainstem and keep the meninges intact. Other landmarks are visible: sternomastoid and digastric muscles, facial nerve, external auditory canal (EAC) (TIF 2897 kb) [file 415_2020_9960_MOESM7_ESM.tif]
